# Cyclic Nucleotide-Directed Protein Kinases in Cardiovascular Inflammation and Growth

**DOI:** 10.3390/jcdd5010006

**Published:** 2018-01-23

**Authors:** Nathan A. Holland, Jake T. Francisco, Sean C. Johnson, Joshua S. Morgan, Troy J. Dennis, Nishitha R. Gadireddy, David A. Tulis

**Affiliations:** Department of Physiology, Brody School of Medicine, East Carolina University, 600 Moye Boulevard, Greenville, NC 27834, USA; hollandn17@ecu.edu (N.A.H.); franciscoj13@students.ecu.edu (J.T.F.); johnsonse12@students.ecu.edu (S.C.J.); morganjo15@students.ecu.edu (J.S.M.); dennist15@students.ecu.edu (T.J.D.); gadireddyn16@students.ecu.edu (N.R.G.)

**Keywords:** cyclic nucleotide, G protein-coupled receptor, interleukin 6, myocardial infarction, inflammation, protease-activated receptor, protein kinase, Smad3, Stat3, vascular smooth muscle

## Abstract

Cardiovascular disease (CVD), including myocardial infarction (MI) and peripheral or coronary artery disease (PAD, CAD), remains the number one killer of individuals in the United States and worldwide, accounting for nearly 18 million (>30%) global deaths annually. Despite considerable basic science and clinical investigation aimed at identifying key etiologic components of and potential therapeutic targets for CVD, the number of individuals afflicted with these dreaded diseases continues to rise. Of the many biochemical, molecular, and cellular elements and processes characterized to date that have potential to control foundational facets of CVD, the multifaceted cyclic nucleotide pathways continue to be of primary basic science and clinical interest. Cyclic adenosine monophosphate (cyclic AMP) and cyclic guanosine monophosphate (cyclic GMP) and their plethora of downstream protein kinase effectors serve ubiquitous roles not only in cardiovascular homeostasis but also in the pathogenesis of CVD. Already a major target for clinical pharmacotherapy for CVD as well as other pathologies, novel and potentially clinically appealing actions of cyclic nucleotides and their downstream targets are still being discovered. With this in mind, this review article focuses on our current state of knowledge of the cyclic nucleotide-driven serine (Ser)/threonine (Thr) protein kinases in CVD with particular emphasis on cyclic AMP-dependent protein kinase (PKA) and cyclic GMP-dependent protein kinase (PKG). Attention is given to the regulatory interactions of these kinases with inflammatory components including interleukin 6 signals, with G protein-coupled receptor and growth factor signals, and with growth and synthetic transcriptional platforms underlying CVD pathogenesis. This article concludes with a brief discussion of potential future directions and highlights the importance for continued basic science and clinical study of cyclic nucleotide-directed protein kinases as emerging and crucial controllers of cardiac and vascular disease pathologies.

## 1. Introduction

Cardiovascular disease (CVD) is a complex and multifaceted class of diseases or disorders of the heart and/or blood vessels and constitutes the number one killer of individuals in the United States [1] and worldwide, accounting for ~18 million (>30%) global deaths annually [2]. Of the many forms of CVD including arterial or venous thromboses, myocarditis, hypertension and valve dysfunction, myocardial infarction (MI) and peripheral or coronary artery disease (PAD, CAD, respectively) represent two of the most significant and account for the vast majority of CVD-related deaths [1]. Notwithstanding significant advances in our understanding of many of the foundational elements underlying CVD realized through extensive basic and clinical investigation, precise and fully effective therapeutic targets have yet to be identified and the prevalence of CVD continues to rise and is expected to afflict ~44% of the US population by the year 2030 [1]. In parallel, the economic burden of CVD, currently estimated over $316 billion per year for direct and indirect costs in the United States alone, is anticipated to surpass $900 billion by 2030 [1]. Undoubtedly, the health and economic impacts of CVD are of utmost significance, and in turn, identification and characterization of key underpinnings in the pathogenesis of CVD in an effort to discern potential therapeutic effectors and/or strategies is most warranted.

In this light, over many years a plethora of bioactive elements and signaling processes has been identified as serving a wide variety of roles in cardiovascular physiology and disease. Of these, the ubiquitous and multifunctional cyclic nucleotide second messenger systems, comprised primarily of purine 3′,5′-cyclic adenosine monophosphate (cyclic AMP) and purine 3′,5′-cyclic guanosine monophosphate (cyclic GMP) and their downstream serine (Ser)/threonine (Thr) protein kinase effectors, serve a multitude of roles in normal vessel physiology and homeostasis and also in the pathogenesis of cardiac and vascular disorders [3]. Of particular interest in cardiovascular tissues are members of the cyclic nucleotide-driven AGC family of Ser/Thr protein kinases: cyclic AMP-dependent protein kinase (PKA), cyclic GMP-dependent protein kinase (PKG), and calcium (Ca^2+^)-activated phospholipid-dependent protein kinase C (PKC) [4]. These robust signaling molecules serve extensive roles in many homeostatic and pathologic processes. Moreover, considering the documented promiscuity and cross-talk amongst protein kinase family members [5,6,7,8] and interactions with associated kinases including protein kinase B (PKB/Akt), protein kinase D (PKD), and adenosine monophosphate (AMP)-activated protein kinase (AMPK), the expansion of the biological impact of these kinases is of potential clinical importance in CVD. This review article focuses on our current state of knowledge of the cyclic nucleotide-driven Ser/Thr protein kinases and their related kinase effectors as emerging and important controllers of cardiac and vascular disease pathology [3].

Many functional processes contribute to the development and/or maintenance of CVD depending on the exact nature of the disease or disorder. As mentioned, CVD comprises a large and diverse class, with MI and arterial diseases considered the most critical and clinically significant. In the causation of MI and arterial disease pathologies, primary functional events associated with tissue perfusion include disruption of blood flow or ischemia with ensuing hypoxia and localized acidosis and complications associated with re-establishment of blood flow or re-perfusion injury. Additional contributors to these pathologies can include extensive tissue inflammation, cell necrosis and apoptosis/necroptosis with compromised tissue function, and loss of a quiescent homeostatic phenotype with onset of aberrant cellular growth/wound healing leading to adverse tissue remodeling [6,9,10]. At the biochemical and molecular level, a host of mechanisms underlie these functional changes and include, notably, an array of immune and inflammatory responses, membrane receptor-mediated signals including G protein-coupled receptor (GPCR) and growth factor pathways, and transcriptional platforms for synthetic and proliferative proteins. In this respect, this review article discusses the biological significance of inflammatory interleukin-6 (IL-6) signaling, acidosis-sensitive GPCRs and protease-activated receptors (PARs), synthetic transforming growth factor-β (TGF-β) and its primary Smad-dependent processes, and a transcriptional Smad/FoxO relationship during CVD. Particular attention is then given to the regulatory influence of cyclic nucleotide-directed Ser/Thr protein kinases on these processes as foundational elements of cardiac and vascular pathology. This review concludes with a short synopsis of key findings and highlights the importance for continued basic science and clinical study of cyclic nucleotide-directed protein kinases in CVD.

## 2. Cyclic Nucleotides and Cyclic Nucleotide-Directed Protein Kinases

**Figure 1:**
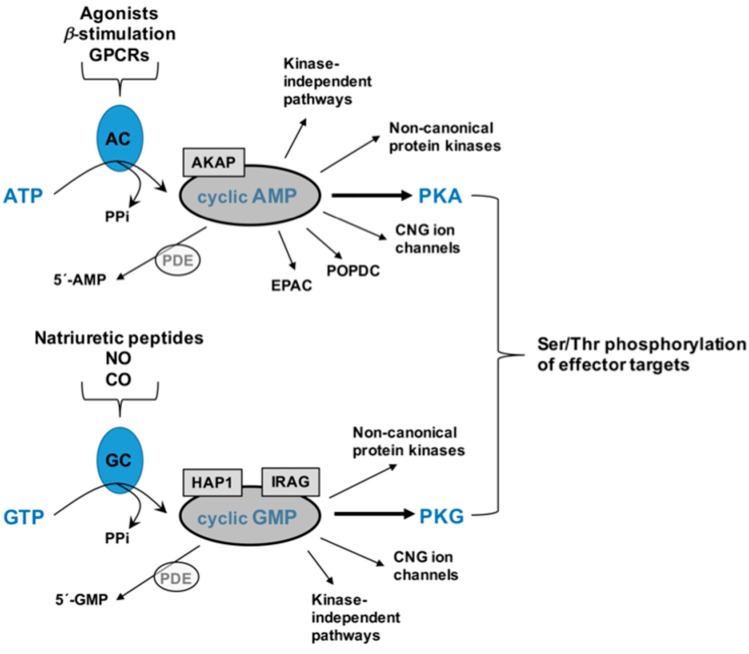
Schematic of cyclic adenosine monophosphate (cyclic AMP) and cyclic guanosine monophosphate (cyclic GMP) signaling. Following activation of adenylate cyclase (AC) by upstream processes including direct ligand agonism, B-adrenergic stimulation, or by stimulatory G protein-coupled receptors (GPCRs), adenosine triphosphate (ATP) is dephosphorylated to yield cyclic AMP and pyrophosphate (PPi). In similar fashion, stimulation of membrane-bound or soluble guanylate cyclase (GC) by natriuretic peptides or gaseous ligands nitric oxide (NO) and/or carbon monoxide (CO), GTP is dephosphorylated to yield cyclic GMP and PPi. Persistence of cyclic nucleotide signaling can be governed by the presence of scaffolding proteins including A-kinase anchoring protein for cyclic AMP or IP_3_ receptor-associated cGMP kinase substrate (IRAG) and Huntingtin-associated protein 1 (HAP1) for cyclic GMP, and by degradation into inactive 5′-monophosphates by a family of phosphodiesterases (PDEs). Cyclic AMP can operate through kinase-independent pathways, through binding to cyclic nucleotide-gated (CNG) ion channels or Popeye domain-containing proteins (POPDC), via exchange proteins directly activated by cyclic AMP (EPAC), through non-canonical protein kinases or by activation of PKA. In like manner, cyclic GMP can signal through kinase-independent pathways, by binding to CNG ion channels, through non-canonical protein kinases or via PKG. The predominant protein kinases for cyclic AMP and cyclic GMP, PKA, and PKG, can then stimulate Ser/Thr residues on many diverse downstream effector targets to help control normal physiology and homeostasis as well as wide-ranging pathophysiological processes in cardiac and vascular tissues.

As mentioned, the purine-based second messengers cyclic AMP and cyclic GMP are firmly established as essential modulators of wide-ranging cellular functions in mammalian tissues including those in the cardiovascular system. Detailed biomolecular mechanisms for the generation of cyclic AMP and cyclic GMP have been previously described [7,10,11,12]. In brief, synthesis of cyclic AMP occurs via multiple processes including stimulation of adenylate cyclase (AC) by direct ligand agonism, by β-adrenergic induction, or by GPCRs coupled to stimulatory G proteins (G_s_). Following AC stimulation, dephosphorylation of adenosine triphosphate (ATP) yields cyclic AMP and pyrophosphate (PPi). In like fashion, cyclic GMP is synthesized via activation of guanylate cyclase (GC), which can occur by natriuretic peptides (NPs) which activate particulate, membrane-bound GC, or by gaseous ligands such as nitric oxide (NO) or carbon monoxide (CO) which activate soluble GC (sGC). Activated GC then dephosphorylates guanosine triphosphate (GTP) to yield cyclic GMP and PPi. Following synthesis, cyclic AMP and cyclic GMP predominantly exert their effects through respective Ser/Thr AGC kinase family members PKA and PKG [4,5,7,10]. Cyclic AMP can also operate through alternate non-canonical kinase-directed pathways [5], through direct ion current modulation via cyclic nucleotide-gated (CNG) ion channels [13], via binding to Popeye domain-containing (POPDC) proteins [14,15], or through exchange proteins directly activated by cyclic AMP (EPAC) [16,17]. Similarly, cyclic GMP can activate CNG ion channels [13], can act on alternate non-canonical kinases besides PKG and can have kinase-independent effects as well [9]. Cyclic nucleotide signaling can be largely governed by internal localization via scaffolding proteins such as A-kinase anchoring protein for cyclic AMP [18] and inositol 1,4,5-triphosphate (IP_3_) receptor-associated cGMP kinase substrate (IRAG) and Huntingtin-associated protein 1 (HAP1) for cyclic GMP [19,20]. Lastly, persistence of cyclic AMP and cyclic GMP signals is largely governed by specific phosphodiesterases (PDEs), which cleave their phosphodiester bonds and degrade them into inactive 5′-monophosphates [21]. The schematic in Figure 1 depicts primary routes for synthesis of cyclic AMP and cyclic GMP, their main regulatory modulators, and their respective activation of downstream targets including protein kinase pathways.

Protein kinases have the capacity to serve in a host of physiological and pathophysiological processes and represent one of the most diverse and ubiquitous families in the human genome, constituting ~2% of human genes with over 500 human protein kinases identified thus far [5,22]. Through reversible phosphotransferase-mediated, site-specific (Ser/Thr) phosphorylation of effector proteins, PKA and PKG provoke robust signal transduction cascades with the capacity to control a myriad of intracellular processes. Comprehensive reviews on the mechanisms of action of these protein kinases and others have been published [23,24] and herein only a brief synopsis is provided. In sum, ATP binds to an active site in a conserved catalytic domain (~250 amino acids in length) located between one lobe of N-terminal β-sheets and a second lobe of C-terminal α-helices [25]. Following binding, a set of conserved residues in the catalytic domain transfers the terminal γ-phosphate of ATP to the hydroxyl oxygen of the receiving residue (Ser/Thr) on the target [23,26], after which substrate is released, ADP is removed and phosphorylation-driven activation or inactivation of the downstream effector ensues. This kinase-driven, post-translational, phosphorylation-specific modification of effector proteins then dictates enzyme and protein expression and/or activities and downstream functions including those elemental to cardiac and vascular disease or dysfunction. Uniquely, despite this common mechanism across diverse protein kinases, kinase specificity is imparted by differences in hydrophobicity of surface residues, unique aspects of the active catalytic site and differential kinetics of ATP binding, the overall charge of the enzyme, and presence or absence of anchoring or scaffolding proteins and other accessory proteins along with sub-cellular localization of the kinase.

A caveat must be mentioned when discussing the kinase-mediated impact on target proteins. While Ser/Thr kinases (as well as Tyr kinases) typically act on their preferred residue, they are also attracted to residues flanking their canonical phosphoacceptor site. Among similar substrate family members, the catalytic cleft of these kinases has the capacity to interact with common recognition sequences adjacent to their preferred substrate (Ser/Thr, Tyr), thereby reducing kinase specificity and permitting ‘promiscuous’ kinase signal transduction. Our research team and others have documented promiscuity and signaling cross-talk for not only the cyclic nucleotide-driven kinases but also for their upstream modes of activation including the second messengers themselves [5,6,7,8,9,10,27,28,29,30,31,32,33]. In this regard, kinase ‘cross-talk’ affords broad impact of upstream kinase activation but also lends difficulty in determining discrete downstream signaling pathways and effector targets of kinase-mediated events.

It warrants brief mention that in opposition to the Ser/Thr protein kinases a family of dephosphorylating Ser/Thr protein phosphatases (PPs) exists that serves to maintain phosphorylative balance [34,35]. Removal of a phosphate group or groups from Ser/Thr residues by PPs curbs phosphorylation-mediated events in Ser/Thr kinase-targeted proteins and helps to moderate kinase-driven processes. Interestingly, only about 30 Ser/Thr PPs have been identified in the human genome (compared to >400 Ser/Thr protein kinases [5,22,34,35]), which is attributed to their unique combination of homoenzymes from shared catalytic subunits and their large number of regulatory subunits [35]. With respect to cardiovascular physiology and disease, Ser/Thr PPs in addition to their complementary Ser/Thr kinases must be considered for full evaluation of the phosphorylative balance and its potential therapeutic utility [8,36].

From a clinical perspective, mutations in Ser/Thr protein kinases have been linked to human diseases, and protein kinases currently represent a large percentage of all putative protein drug targets [37,38,39,40]. In fact, many protein kinases presently serve as discrete targets for use in precision medicine for cardiac and vascular diseases [3] and they likely represent the next major drug development target for diseases and disorders of the cardiovascular system (and others) in the next century [41,42].

## 3. Vascular Physiology & Pathology

**Figure 2:**
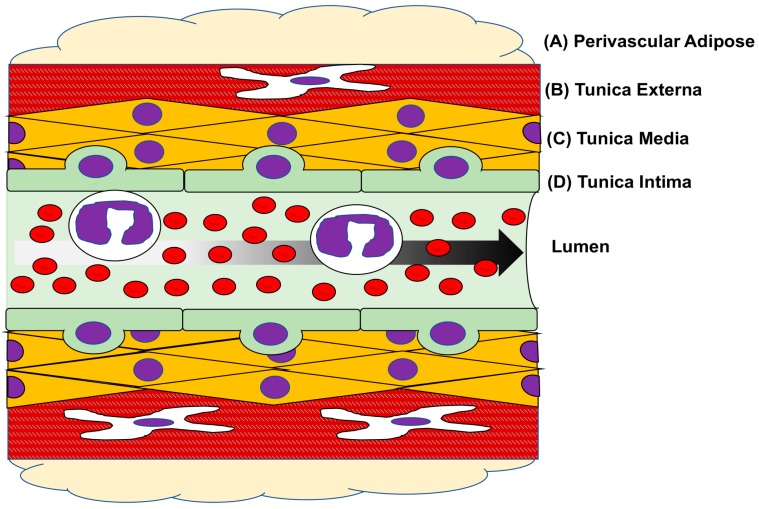
Layers of a blood vessel. Cartoon cross-sectional image of a blood vessel with major layers and cell types depicted. The outermost perivascular fat (**A**) lends support and anchoring for the vessel as well as mediates adipocyte production and influences cellular metabolism. The outermost layer of the vessel proper, the tunica adventitia (**B**); is largely a structural layer of the vessel wall and is comprised of extracellular matrix (ECM) containing resident immune cells, an internal vascular supply (vasa vasorum), sparse nerve endings and fibroblasts. The majority of the arterial vessel wall is comprised of the tunica media (**C**); mostly vascular smooth muscle cells (VSMCs) and ECM. Medial VSMCs are responsible for vasoconstriction and relaxation (i.e., vessel tone) that controls luminal blood flow. The innermost layer of the blood vessel is the tunica intima (**D**) and is comprised of a single layer of vascular endothelial cells (VECs) that surround the lumen of the vessel, that form a critical interface between flowing blood and the vessel wall, and that communicate with the underlying VSMCs to help regulate tone and direct inflammatory responses. Arrow indicates direction of luminal blood flow.

Excellent comprehensive reviews have been published recently that highlight important aspects of blood vessel anatomy and function under homeostatic and pathologic conditions [7,9,43]. In sum, normal arterial anatomy consists of three primary layers: tunica intima, tunica media and tunica externa. A single layer of vascular endothelial cells (VEC) encircle the blood-containing lumen and constitutes the tunica intima. Intimal VECs are normally exposed to laminar shear stress and provide an important interface between flowing thrombogenic blood and the blood vessel wall [43]. In this capacity VECs are responsible for secreting bioactive substances, including the vasodilators NO, CO and prostaglandin I_2_ (PGI_2_) and the vasoconstrictors thromboxane A_2_ (TXA_2_) and angiotensin II (Ang II), that communicate with the underlying vascular smooth muscle (VSM) through the basement membrane in order to control vascular tone [43]. The tunica media, the blood vessel inner layer, contains vascular smooth muscle cells (VSMCs) as well as structural collagen and elastin and is responsible for maintaining normal vascular contraction and dilation. This vessel reactivity, in turn, controls localized intravascular pressures and tissue perfusion [44]. Medial VSMCs are known to be much more plastic than other vessel wall cells because they handle many functions such as contraction and dilation as well as proliferation and extracellular matrix (ECM) synthesis [44]. The outward component of the vessel wall is the tunica externa or adventitia which contains sparse fibroblasts and VSMCs, a vasa vasorum blood supply, and local nerve endings and inflammatory cells spaced throughout the supporting connective tissue [43]. Perivascular adipose tissue located on the outside of the tunica adventitia plays a role in support and anchoring of the vessel yet has also been suggested to serve a role in energy metabolism, regulation of vascular tone, the release of adipokines, and in the storage of free fatty acids and triglycerides [43]. A schematic depicting these key elements in a cross-section of a blood vessel wall is shown in Figure 2.

The predominant overall function of the arterial vasculature is to provide blood flow and nutrients to essential downstream tissues in order to ensure their proper function under homeostatic as well as abnormal conditions. In return, the venous system serves as the conduit for removal of metabolic byproducts and wastes including carbon dioxide for elimination from the body. Based on the amount and functionality of local VSM, most blood vessels have ability to constrict or relax as needed in order to adequately control blood flow and, in turn, to properly supply tissues with blood and nutrients including oxygen as needed per local metabolic demand. In this light, the vasculature provides a basal state of tonic contraction termed vascular tone (or ‘myogenic tone’ if derived from the VSM itself). In brief, to first summarize vessel contraction, extracellular calcium entry (via voltage-gated, ligand-gated, and/or stretch-activated Ca^2+^ channels) induces intracellular Ca^2+^ levels to rise (via the release of Ca^2+^ from intracellular stores). This elevated intracellular Ca^2+^ then binds to calmodulin and sequentially activates cytosolic Ser/Thr myosin light chain kinase, which then phosphorylates regulatory myosin light chain and activates myosin ATPase activity. This ATPase then initiates actin-myosin cross-bridge formation and cycling and the establishment/maintenance of vessel contraction [10,11]. For vessel relaxation, removal of intracellular Ca^2+^ is the first step and this can occur via re-sequestration back into intracellular stores and/or removal from the cell by Ca^2+^ channels. Following the Law of Mass Action, Ca^2+^ unbinds from calmodulin, myosin light chain kinase activity decreases, myosin phosphatase dephosphorylates myosin light chains, which in turn reduces myosin ATPase activity and muscle tension is decreased. Vascular tone can be regulated by extrinsic (neurohumoral elements, CNS innervation) and intrinsic (VSM-derived, myogenic) elements and so can be determined by numerous influences including competing vasoconstrictor and vasodilator factors and local metabolic demand of downstream tissues in organ- and tissue-specific fashion. Low-level vascular tone results from numerous and differential states of cross-bridge formation that can develop which leads to various contractile states of the VSM including notably a basal steady-state level of contraction (tone). Regarding cyclic nucleotide control of vascular tone, both cyclic AMP/PKA and cyclic GMP/PKG can operate via several mechanisms to reduce intracellular Ca^2+^, to inhibit myosin light chain phosphorylation, and to stimulate Ca^2+^-activated potassium channels and promote hyperpolarization, all resulting in reduction in vascular contractility and tone and promotion of vessel relaxation (loss of tone) [10,11].

Despite the essential role that our circulatory system plays in normal cardiovascular health, pathological conditions such as vascular disease, dysfunction or injury constitute the number one contributor to CVD [2]. Two primary components in the pathogenesis of vascular disease or injury include overt inflammation of the intimal endothelial lining and abnormal synthesis and growth of medial VSMCs [9,45,46]. In diseased or dysfunctional VECs, production and release of inflammatory cytokines along with increased substrate adhesiveness contribute to the recruitment of leukocytes, which adhere to the (activated) cells, transmigrate and provoke an inflammatory response [47]. In this process, increased expression of vascular cell adhesion molecules, cytokines, and chemokines is essential for the VEC-leukocyte interaction and subsequent inflammation; thus, identification and characterization of molecular mediators and events that regulate VEC inflammation and adhesion is critical. In this light, key VEC inflammatory mediators have been identified including E-selectin, ICAM-I, and VCAM-1 that have potential to serve as therapeutic targets to combat VEC inflammation in the context of CVD [48]. Complementing VEC-driven inflammation is the VSM-dependent proliferative, synthetic ‘evolution’ phase of CVD pathogenesis [5,9,10,49]. In response to pathologic insult, VSMCs undergo phenotypic switching from homeostatic and contractile to synthetic, migratory and proliferative. This phenotypic conversion is manifested as reduced contractility and a reorganized architecture complete with medial wall remodeling and stenotic neointima development [9,44,50]. This neointimal growth involves fibroblast and cellular accumulation in the perturbed intimal space that results in excessive synthesis and deposition of ECM components and loss of luminal caliber resulting in altered or occluded blood flow [51]. Although vascular remodeling and neointimal formation initially serve as adaptations they can soon progress into uncontrolled, pathologic and self-perpetuating cascades with severe clinical repercussions.

Vessel wall remodeling and neointimal hyperplasia can provoke and serve as a key component of atherogenesis, the process of the build-up of fats and cholesterol (along with numerous cells, matrix components, etc.) in an occlusive plaque on the inner blood vessel wall. Atherosclerosis is the major cause of adverse cardiovascular events including stroke, MI, and peripheral limb ischemia or claudication [1]. Atherosclerosis is multifactorial and complex, combining elements of inflammation and cellular adhesion, VEC dysfunction, formation of reactive oxygen and nitrogen species and oxidative/nitrosative stresses, foam cell development, VSMC migration and proliferation, enhanced ECM development, and formation and evolution of a stenotic plaque within the lumen. If plaque complication and rupture ensue, thrombus formation and adverse cardiovascular events including MI and/or stroke can rapidly develop.

## 4. Cardiac Physiology & Pathology

The heart is a crucial pump that utilizes the circulatory system to provide the driving force for maintenance of blood pressure and to deliver essential blood flow to target organs. However, any decrement in cardiac function and its ability to serve as a central pump can lead to hypo-perfusion of distal tissues and organs and, in turn, eventuate in organ failure and multi-system dysfunction and ultimately death. In this section, an overview of some general concepts in cardiac physiology and pathology is presented that will be discussed in subsequent sections.

Central to the role of the heart as a pump are several important physiological concepts, and a brief refresher is warranted: chronotropy refers to the generation of pacemaker action potentials in the sinoatrial (SA) node that allow for depolarization of adjacent cells via gap junctions and therefore determines heart rate [52]; SA nodal rate and speed of conduction may by modulated by changes in atrioventricular (AV) nodal depolarization in dromotropy [53]; electrical conduction is converted into mechanical contractility by excitation-contraction coupling via Ca^2+^-induced Ca^2+^ release [54]; cardiac inotropy describes the force of contraction that results from electrical-mechanical coupling and, when combined with chronotropy and dromotropy, determines cardiac output; and following contraction the rate by which the ventricles relax due to sequestration of Ca^2+^ back into internal stores is known as lusitropy [55].

Structurally, the heart is comprised of several primary cell types. Cardiomyocytes comprise the bulk of the organ and are categorized as either specialized conductive cells [56] or as the cells responsible for myocardial contraction [57]. Fibroblasts are responsible for the structure and maintenance of the cardiac ECM which provides support for the structure of the heart as well as contributing to formation of cardiac valves. VECs and VSMCs comprise the blood vessels within the heart, the coronary circulation [58]. Additionally, resident leukocytes such as macrophages, innate lymphoid cells, and mast cells can be found within the myocardium [59]. Each of these cell types and physiological concepts is critical to maintaining healthy cardiac function. Modulation of both cyclic nucleotide-directed protein kinase signaling and inflammatory processes can be exploited clinically to rescue cardiovascular function in the presence of a pathological state or exacerbation of disease processes such as in myocarditis, myocardial infarction, ischemia-reperfusion (I/R) injury, and the development of heart failure (HF) [3].

Epidemiologic studies indicate CVD as the leading cause of morbidity and mortality in the United States with an estimated 1,255,000 new or recurrent events of myocardial infarction (MI) occur per year [60]. This review will focus primarily on pathologies and sequela related to MI including I/R injury as well as HF due to adverse cardiac remodeling following MI. Current medical opinion divides MI into two distinct categories separated by their underlying etiology, Type 1 and Type 2 [61]. Type 1, or spontaneous, MI is considered the prototypical example of an infarction and is the most common type of MI [62]. As discussed above in vascular pathology, ischemia occurs when a vessel becomes occluded by either a thrombus or less often by an embolus. Commonly, luminal blockages of a coronary artery result paradoxically from normal wound healing and clotting responses gone awry. Coronary thrombosis occurs when a complicated atherosclerotic plaque spontaneously ruptures, and the initial recruitment of platelets to the site of injury begins to block the already narrowed arterial lumen. Ultimately, blood flow to distal portions of the myocardium becomes obstructed.

Although the most common pathological mechanism for initiation of MI, rupture of atherosclerotic plaques are not the only means of inducing myocardial ischemia or MI. When oxygen demand outpaces oxygen supply, such as during strenuous exercise [63] or during coronary vasospasm [64], injury can occur. Myocardial oxygen supply-demand mismatch in the absence of coronary thrombosis is classified as a Type 2 MI and is another leading cause of MI. It is important to note that in Type 1 or Type 2 MI, total luminal occlusion of a coronary artery is not required to induce ischemia because any decrease in coronary flow resulting in inadequate oxygen distribution will result in ischemia and if unresolved ultimately cardiomyocyte death [62]. Although Types 1 and 2 MI are the most common etiologies, there exist several classifications relevant to clinical discrimination of MI. Type 3 MI is an entirely clinical subdivision and reflects sudden cardiac death of unknown etiology, but acute myocardial ischemia is strongly suspected [65]. The gold-standard treatment for Type 1 and Type 2 infarcts is reperfusion by percutaneous coronary intervention (PCI), thrombolysis, or coronary artery bypass grafting (CABG) which has led to two new classifications of Types 4 and 5 MI and indicate iatrogenic origins. Type 4 MI results from PCI or stent placement and involves myocardial ischemia that occurs during the procedure or secondary to vessel restenosis [63,66]. Type 5 MI is very similar to Type 4 except that ischemic complications are secondary to CABG. While most Types 4 and 5 infarcts are due to technical failure that limits resolution of ischemia, some are associated with successful intervention in the face of an excessive inflammatory response to reperfusion.

**Figure 3:**
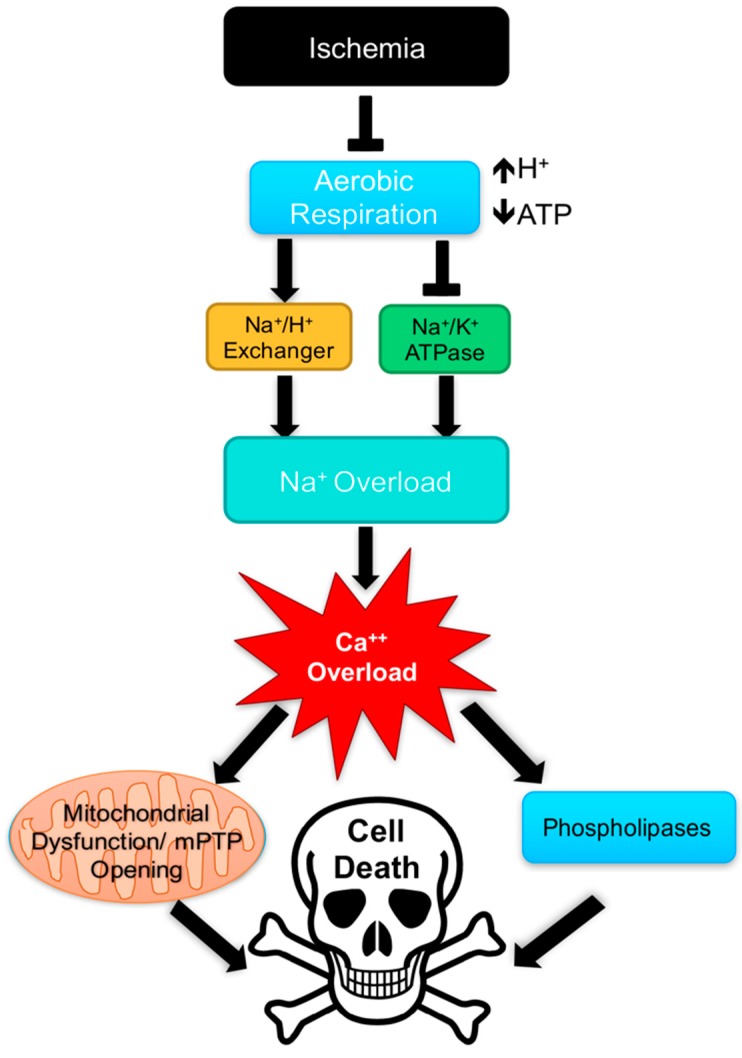
Overview of mechanisms contributing to cell death following ischemia. Ischemia results in ion channel dysfunction following decreased production of ATP and increased hydrogen ion (H+) production leading to cellular acidosis. Ultimately excessive intracellular calcium (Ca^2+^) triggers activation of phospholipases and the opening of the mitochondrial permeability transition pore (mPTP) in turn inducing cell death.

Ischemia is a condition whereby inadequate blood flow results in inadequate oxygen supply [67]. Mechanisms that ultimately lead to cardiomyocyte death are tied strongly to cellular susceptibility to hypoxia. The myocardium has an incredibly high metabolic demand: making delivery of oxygen by way of the coronary arteries crucial in support of normal cardiac function. Occlusion of one or more coronary arteries leads to ischemia in areas distal to the blockage and, in turn, compromises tissue metabolism and function. Thus, ischemia leads to hypoxic injury in distal tissue, which if left unresolved eventuates in cell/tissue death known as infarct [62]. Hypoxic injury to the myocardium results from an inability to generate sufficient ATP via oxidative phosphorylation causing a shift to anaerobic glycolysis [62]. Anaerobic glycolysis results in intracellular acidosis from the accumulation of intracellular hydrogen ions (H^+^), thereby disturbing the sodium (Na^+^)/H^+^ exchanger [68]. Depletion of available ATP inactivates Na^+^/potassium (K^+^) ATPase [69]. The combined ionic disturbances result in Na^+^ overload. As a result, Na^+^-Ca^2+^ exchanger attempts to compensate for the ionic disturbance by pumping Ca^2+^ into the cytoplasm and Na^+^ out of the cell. However, the intracellular Ca^2+^ overload induces cardiomyocyte death [62,64,68,69]. The cytosolic oversaturation with Na^+^ or Ca^2+^ results in increased cytoplasmic osmolality resulting in cellular edema [64]. Excess Ca^2+^ uptake by cardiac mitochondria induces opening of the mitochondrial permeability transition pore (mPTP) leading to mitochondrial lysis, the release of cytochrome C, and induction of apoptosis [70,71,72]. Intracellular Ca^2+^ concentration also activates phospholipases that serve to degrade cardiomyocyte cell membranes [68,69]. The cascade of events following an initial ischemic insult, as highlighted above, contribute to cell/tissue death and ultimately the condition of MI. An overview of some of these key elements that serve as foundations for cardiac dysfunction and disease following an ischemic episode is shown in Figure 3.

To salvage myocardial tissue and rescue normal cardiac function it is imperative to rapidly restore coronary blood flow following onset of cardiac dysfunction and/or MI. Clinically, blood flow can be restored through medical interventions such as thrombolytic therapy, PCI, or CABG [73,74]. The goal of MI treatment, restoration of blood flow, however can paradoxically result in further myocardial injury, known as reperfusion injury [68]. Reperfusion injury broadly encompasses several adverse events including arrhythmia, myocardial stunning, and microvascular damage [64,68,69,75,76]. Although the manifestations of reperfusion injury are diverse, in many respects the underlying mechanisms mirror many of the processes witnessed in primary ischemic injury. Complicating the issue of revascularization and reperfusion is the tendency for the aberrant and pathological proliferation of coronary artery VSMCs leading to vascular restenosis through inflammatory processes and growth/remodeling [77,78]. As will be discussed below, data generated over the past decade has also strongly implicated regulatory roles for cyclic nucleotides and their multifunctional protein kinases in these cardiac pathologies [31,36,79,80,81].

Reactive oxygen species (ROS) play a fundamental role in mediating cardiac reperfusion injury. Surges of oxygen occurring with the restoration of blood flow generate superoxide anion and/or peroxynitrite by cardiomyocyte mitochondria [64,82]. The impact of ROS on reperfusion injury are varied and involve triggering: the activity of protein kinases and subsequent pathways, peroxidation of lipid membranes, apoptosis, and dysfunction in Ca^2+^ handling [82,83,84]. Ca^2+^ handling in reperfusion injury is influenced by cytosolic influxes in Na^+^. Exchange of Na^+^ for Ca^2+^ leads to high cytosolic Ca^2+^, and the overload of Ca^2+^ leads to rigor-type contracture of the myocardium. Rigor-type contraction contributes to the development of myocardial dysfunction. As observed in ischemia, fluctuations in Ca^2+^ released from the sarcoplasmic reticulum stimulate the opening of the mPTP [70,71,72]. Once the mPTP opens the ionic gradient required for synthesis of ATP rapidly dissipates and water can flood into mitochondria causing swelling [64] and rupture of the mitochondria which triggers apoptosis [68].

Another critical component of reperfusion injury results from leukocyte trafficking to the area of injury which occurs shortly after re-establishment of blood flow [64]. Initially, during reperfusion neutrophils move to areas of ischemia to phagocytose dead tissue, releasing cytokines to further mediate the immune response [64,85,86]. Factors secreted by neutrophils which include ROS, cytokines, and chemokines which promote inflammation may also damage previously viable tissue [68]. Neutrophils obstruct post-capillary venules contributing to microvascular dysfunction [85] which is thought to mediate no-reflow phenomenon associated with reperfusion injury [68,85,87]. Platelets recruited to sites of injury may also contribute to reperfusion injury and microvascular dysfunction by secreting vasoactive thromboxane A_2_ and 5-HT. Infiltrating monocytes to the zone of infarction further mediate the inflammatory response to ischemia and reperfusion releasing proteases capable of infarct expansion through proteolysis [68,69,85]. Restoration of flow is required to re-establish oxygenation, so ways to reduce the attendant inflammatory response constitute a significant area of interest in improving recovery from acute ischemic injury.

**Figure 4:**
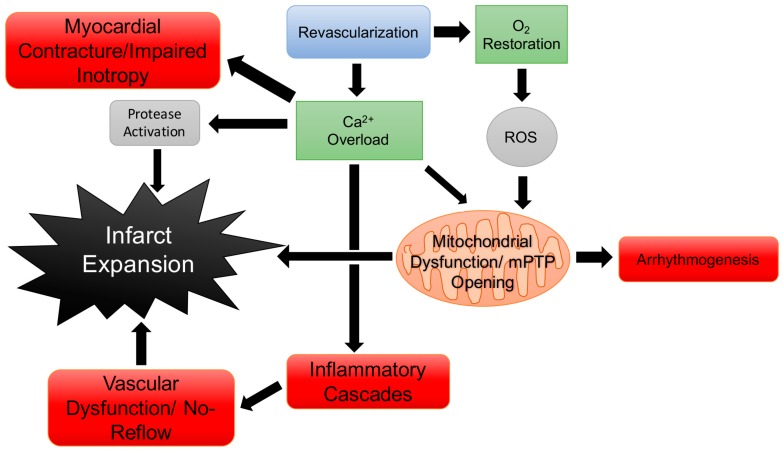
Overview of mechanisms contributing to reperfusion injury. Reperfusion injury results following revascularization and restoration of blood flow to the ischemic myocardium. As with ischemic injury intracellular calcium (Ca^2+^) overload leads to increased myocardial injury through multiple pathways. Reactive oxygen species (ROS) also contribute to myocardial injury in turn promoting inflammation and altering mitochondrial function.

Following MI the most significant predictor the development of HF is infarct size [88] determined by the death of cardiomyocytes from ischemia or reperfusion injury. Therefore, mitigation of injury size after MI is of chief concern for patient prognosis [89]. The homogeneous cell death pathways triggered by MI lead to release of pro-inflammatory cytokines and danger signals [90], which activate the immune system to respond to the initial cardiac insult clearing the infarcted area of cellular and ECM debris. The initial injury response gives way to a reparative response partially mediated by TGF-β/Smad signaling [91,92], culminating in the formation of a non-contractile scar that significantly diminishes cardiac inotropic capacity and subsequently compromises cardiac output leading to decompensated HF. A cartoon showing an overview of cellular processes during reperfusion and ensuing injury is shown in Figure 4.

## 5. Inflammation & IL-6 Signaling

Inflammation can be broadly defined as a response to stress or injury by the immune system, and the inflammatory response results from complex interactions between immune cells such as leukocytes and molecular mediators such as cytokines and chemokines and the damaged or infected cell. Inflammation serves as a host defense process that ensures timely and adequate disposal of dead or dying cells and/or invading pathogens thereby leading to repair of damaged tissues and paving the way for repair and restoration of tissue and organ function. As discussed, inflammation plays a crucial role in response to bodily insult and is critical to injury repair, particularly in cardiac and vascular tissues. However, perturbations in the typical inflammatory response or unresolved or uncontrolled inflammation plays a pivotal role in the etiology of CVD including but not limited to atherosclerosis [93], MI [94], I/R injury [68], vasculitis [95], neointimal hyperplasia and restenosis [96], hypertension [97], and HF [98].

Cytokines serve as essential proteins that play critical roles in cell-cell communication in response to injury and in the initiation, maintenance, and resolution of inflammation. There is a strong association with elevated levels of circulating cytokines and CVD. In humans with CAD, it has been shown that elevated serum concentrations of IL-1β, TNFα, IL-8, and IL-27 are present during events of unstable angina or are elevated in exacerbations of HF [99,100]. IL-1β and TNFα in addition to IL-18 are responsible for enhanced surface expression of the selectins VCAM-1 and ICAM-1 [101,102,103], which indicate endothelial activation due to inflammation and may play roles in vascular changes associated with particle exposure and no-reflow phenomenon associated with reperfusion following ischemia [85,104]. IL-1 family cytokines including IL-18 have also been shown to contribute to LV dysfunction [105,106]. TNFα is associated with cardio-depression and dysfunction following MI [107]. IL-2 is a pro-inflammatory cytokine key to T cell differentiation, thereby promoting helper Th1 and Th2 while inhibiting Th17 and T follicular helper cells [108]. Infusion of exogenous IL-2 has been shown to induce heart failure and myocarditis [109,110]. Considering the overall impact on cardiovascular dysfunction directly attributed to pro-inflammatory cytokines in addition to secondary damage from leukocyte recruitment, cytokines play key roles in mediating cardiovascular dysfunction and expansion of cardiac injury.

Interleukin-6 (IL-6) is a pleiotropic cytokine with both pro-inflammatory and anti-inflammatory properties [111,112,113]. Increased levels of pro-inflammatory IL-6 are associated with atherosclerosis [93], vasculitis [114], MI [94] and HF [115]. Despite the apparent connection between IL-6 and CVD, the IL-6 receptor is only expressed on hepatocytes and in certain leukocytes [116]. The process when IL-6 binds to a membrane-bound IL-6 receptor to induce signal transduction is referred to as classical IL-6 signaling. This limited expression of the IL-6 receptor has traditionally been considered a constraint to the biological significance of classical IL-6 signaling; however, an alternative mechanism by which IL-6 may act directly on the cells of the cardiovascular system has been identified as IL-6 trans-signaling and through this mechanism the biological impact of IL-6 processes are significantly expanded.

Interleukin-6 trans-signaling may serve to override negative feedback mechanisms of classical IL-6 signals in addition to allowing cells without the IL-6 receptor to respond to IL-6. Adding to its biological impact, IL-6 trans-signaling has been implicated in the pathogenesis of conditions including atherosclerosis [117], hemorrhagic trauma [118], and inflammation-based CVD [119]. Over the past decades, much investigation has focused on the role of IL-6 in the pathogenesis of CVD; however, with the recently discovered soluble IL-6 receptor and its trans-signaling mechanism interest has returned to investigating IL-6 in CVD. As a result, this review will focus primarily on the roles IL-6 and trans-signaling in the development or abrogation of CVD.

**Figure 5:**
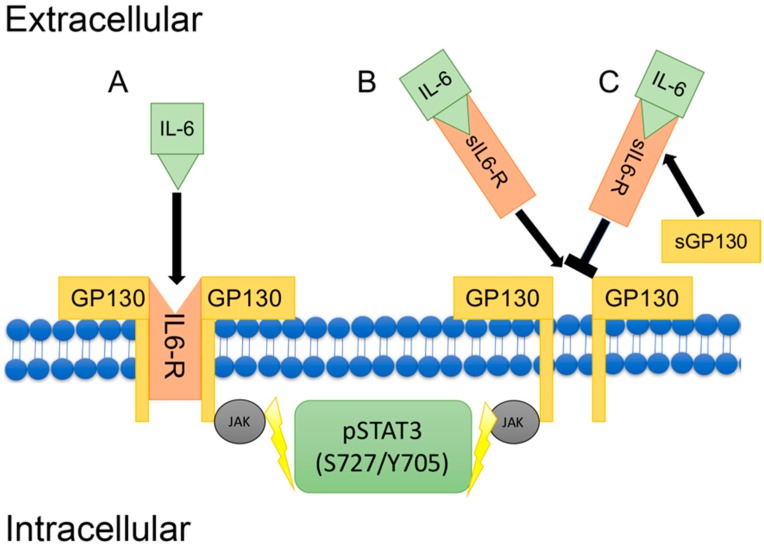
Classical versus interleukin-6/(IL-6) trans-signaling. A simplified schematic depicts classical IL-6 signaling whereby IL-6 binds to membrane-bound IL-6 receptor (IL-6R) initiating signal transduction via glycoprotein 130 (GP130) to increase intracellular STAT3 via JAK (**A**); Alternatively, in (**B**) IL-6 trans-signaling occurs whereby IL-6 binds to soluble IL-6 receptor (sIL-6R) which then complexes to membrane-bound GP130/JAK to initiate STAT3 signal transduction. Inhibition of IL-6 trans-signaling can occur by the decoy soluble GP130 (sGP130) (**C**).

The interplay and balance of several soluble factors modulate the ability of IL-6 trans-signaling to exert its effects in cardiovascular tissues: a soluble IL-6 receptor (sIL-6R) or soluble glycoprotein 130 (sgp130) act to promote or inhibit IL-6 trans-signaling, respectively. Over time, cardiac injury induces aberrant increases in IL-6 trans-signaling and in turn, mediates adverse cardiac remodeling and exacerbation of HF. Inhibition of IL-6 signal transduction with a blocking IL-6 receptor antibody in a murine model has been shown to ameliorate left ventricular remodeling following MI [120]. Previous work regarding IL-6 biology has focused specifically on classical IL-6 pathways or has sought to attenuate the effects of IL-6 signaling indiscriminately by blocking both classical and trans-signaling through IL-6 receptor blockade. However, this approach neglects the distinct possibility that classical and trans-signaling may play independent biological roles in the cardiovascular injury response. A schematic depicting mechanisms of classical IL-6 signal transduction and IL-6 trans-signaling is shown in Figure 5.

The balance between IL-6 classical and trans-signaling is crucial to physiological function, whereby they have the capacity to produce divergent effects on inflammation and ensuing pathologies [121]. Both mechanisms of IL-6 signaling activate Janus-Kinase (JAK)/Signal Transducer and Activator of Transcription (STAT) pathways, which primarily lead to the activation of STAT3 [122], which has been shown to have cardioprotective effects by acutely promoting cardiomyocyte survival and compensatory hypertrophy [123]. However, prolonged expression of STAT3 has also been demonstrated to negatively impact cardiac function following MI [124].

Of interest, IL-6 signaling may further modulate cardiovascular function by mediating, or getting mediated by, cyclic nucleotide-driven protein kinases. IL-6 signaling has been implicated in decreased cardiac inotropy in adult rat ventricular myocytes mediated by the cyclic GMP/PKG pathway via IL-6 transcriptional upregulation of iNOS [125]. Although IL-6 may induce impaired myocardial function via PKG signals, chronic activation of β-adrenergic receptors, as occurs in HF, can induce IL-6 expression through cyclic AMP/PKA and the induction of STAT3 through IL-6 may induce cardiomyocyte hypertrophy [126], thereby implicating IL-6 signaling in the progression to decompensated HF. However, it remains unclear whether IL-6 classical or trans-signaling is responsible for these observations.

## 6. Cardiac Physiology/Pathology & Cyclic Nucleotide-Directed Protein Kinases

The complex processes that lead to the development of CVD and particularly MI, I/R injury, can to a certain degree be attenuated or exacerbated by the activity of certain cyclic nucleotide-dependent protein kinases. One such protein kinase responsible for the mitigation of I/R injury is cyclic GMP-driven PKG. Ischemia has been demonstrated to modulate intramyocardial levels of cyclic GMP in multiple animal models, initially increasing cyclic GMP content within the first few minutes of ischemia [127,128]. Furthermore, experimental models of preconditioning prior to an ischemic episode have shown increased myocardial cyclic GMP content compared to tissues exposed to ischemia alone [129]. In the heart, NO is mostly responsible for sGC activation and conversion of GTP to cyclic GMP and subsequent activation of PKG. Ca^2+^ homeostasis can be modulated by PKG interactions with both IP_3_ receptors [130] as well as with ryanodine receptors (RyR) [131]. Additionally, Ca^2+^-sequestering phospholamban can act as a substrate for PKG [132]. Ca^2+^ handling can also be modulated by PKG activity via interaction with L-type Ca^2+^ channels and Ca^2+^-activated potassium channels, which can all negatively influence cardiac inotropy and chronotropy in addition to reducing VSM tone within the coronary circulation. Cardiac inotropy can also be modulated by interactions of PKG with troponin, thereby decreasing the contractile responsiveness to Ca^2+^ [79,133]. The ability of PKG to modulate cardiac ischemia-reperfusion injury through its actions on thromboxane A_2_ receptor and then to inhibit ensuing signal transduction potentially limits platelet activation and aggregation through desensitization [80,134,135], which may ultimately attenuate vascular occlusion due to atherosclerotic rupture or no-reflow phenomenon after reperfusion. Recently and of importance, Frankenreiter and Lukowski and colleagues reported that cyclic GMP and PKG are able to exert cardioprotective effects through stimulation of cardiomyocyte-specific big potassium (BK) channels, demonstrating acute infarct sparring as well as decreased myocardial dysfunction following MI by cyclic GMP/PKG signals [136].

Another critical cyclic nucleotide-directed pathway involved in cardiovascular physiology and pathology involves cyclic AMP-stimulated PKA. As described, PKA is activated by cyclic AMP following its generation from ATP by AC following a variety of mechanisms including catecholamine-mediated stimulation of β-adrenergic receptors [41]. Similar to PKG, PKA has been demonstrated to interact with RyR [137] and L-type Ca^2+^ channels [81,138] to increase cytoplasmic Ca^2+^ concentrations thereby stimulating myocardial contraction while also enhancing Ca^2+^ uptake via phospholamban and increasing cardiac lusitropy [139]. PKA can increase myocardial contraction through phosphorylation of cardiac troponin I (cTnI) inducing accelerated cross-bridge cycling by sensitizing actinomyosin ATPase to Ca^2+^ [140]. Phosphorylation of cardiac myosin binding protein (cMyBP) by PKA induces inotropy by modulating the interaction between thick and thin filaments [141]. Interestingly, hypo-phosphorylation of cMyBP has been demonstrated to be associated with worsening failure HF in animal models [142,143] and in failing hearts in humans [144]. Like PKG, the role of PKA in CVD and particularly MI is multifold and can have direct effects on cardiac function and injury as well as indirect effects that can modulate injury and function which, in many cases, overlap with PKG signaling in apparently antagonistic fashion. While NO ultimately activates downstream PKG and is responsible for decreasing cardiac inotropy, lusitropy, chronopropy, dromotropy, and automaticity, the actions of PKA generally promote cardiac inotropy, lusitropy, chronopropy, dromotropy, and automaticity.

## 7. G Protein-Coupled Receptor Signaling

The family of GPCRs constitutes the broadest and most diverse group of membrane receptors identified in the human genome that has the capacity to control a myriad of cellular functions. The receptor is integrated into the plasma membrane by seven transmembrane loops, and upon binding of an extracellular agonist a conformational change in the receptor occurs. The cytoplasmic carboxyl tail of the receptor interacts with nearby heterotrimeric (α, β, γ) G proteins, GDP (bound to Gα) is replaced by GTP (which activates stimulatory Gα_s_), the β and γ subunits dissociate (remaining as a dimer), and intracellular signaling ensues via Gα_s_-GTP, other Gα subunits, and Gβγ [145]. Both activated Gα subunits and the βγ dimer can interact with numerous membrane proteins to induce broad and diverse signal transduction processes. In fact, activation of a single GPCR can provoke thousands of downstream second messenger signals including commonly AC-mediated cyclic AMP, diacylglycerol and IP_3_, and intracellular Ca^2+^. In opposition to activation by Gα_s_-GTP, stimulation of the inhibitory subunit (_i/o_) of Gα results in inhibition of AC and reduction of cyclic AMP synthesis and downstream PKA signaling [3,7,145]. This complex and highly regulated signal transduction system affords GPCRs the control of innumerable cardiovascular functions and makes GPCRs a critically important target for modern medicinal drugs [146,147,148,149,150,151].

A unique family of acidosis/pH-sensing heterotrimeric GPCRs has been identified and characterized as extracellular proton sensors and is comprised of G protein-coupled receptor 4 (GPR4), T cell death-associated gene 8 (TDAG8 or GPR65), ovarian cancer G protein-coupled receptor 1 (OGR1 or GPR68), and G protein-coupled receptor G2 accumulation (G2A or GPR132) [152,153,154,155,156,157,158]. This family of GPCRs are activated via acidic protonation of histidine residues on the extracellular amino binding domain and signal predominantly through the intracellular subunits Gα_s_ (AC/cyclic AMP stimulation), Gα_q/11_ (PLC/DAG/IP_3_ and Ca^2+^ stimulation), Gα_12/13_ (RhoGEFs/RhoA/Ras stimulation), as well as through the Gβγ subunit (PI3K, GRKs, PLC and Ca^2+^ stimulation) [157]. Obviously, these GPCRs then have the capacity to moderate broad downstream kinases including PKA and PKC as well as MAPKs/ERKs. Cardiovascular tissues and inflammatory cells including leukocytes contain all of these pH-sensing GPCRs, yet interestingly, VECs predominantly express GPR4 [152,158,159,160] while VSM and cardiomyocytes largely express GPR68 [155,157,161].

During deleterious conditions as found in CVD and other disorders, dysregulated and largely glycolytic anaerobic cellular metabolism occurs which, along with impaired blood perfusion and several other processes, causes acidic byproducts to accumulate, in turn acidifying the local tissue microenvironment. Considering that acidosis is a strong cellular stressor, redundant modes exist for the sensing of extracellular acidosis and the facilitation of downstream signaling and modulation of cellular responses. While it has been theorized that acid-sensing GPCRs may play key roles in the detection of extracellular acidosis and/or in the development or maintenance of cardiovascular pathologies, fundamental mechanisms responsible for the ability of these important GPCRs to moderate cardiovascular dysfunction and their potential interaction with cyclic nucleotide-dependent protein kinases remain to be fully elucidated. Recent work in human umbilical vein endothelial cells (HUVECs) and lung microvascular and pulmonary VECs showed that isocapnia (as found in metabolic acidosis) or hypercapnia (such as respiratory acidosis) independently activate GPR4 to induce broad inflammation characterized by significant upregulation of pro-inflammatory genes including members of the CXC and CCL families of cytokines and chemokines, vascular cell adhesion molecules E-selectin, VCAM1 and ICAM1, members of the TNF and NF-*κ*B signaling systems, elements in the prostaglandin-endoperoxidase synthase family, and transcription factor early growth and stress response genes [159,160]. Using Gene Ontology (GO) Enrichment with the GATHER systems approach [162], the families of acid/GPR4-induced genes correlate with immune, defense and inflammatory responses, consistent with the induction of the inflammatory response through acidosis and GPR4 [160]. It has also been determined that acidic pH induces gene expression of the vascular cell adhesion molecules E-selectin, VCAM1 and ICAM1 and that it does so in cyclic AMP/GPR4-dependent fashion [152,157,159,160]. Complementing these observations, additional work by Yang and colleagues [159,160] showed that acidosis and GPR4, alone or in synergy, significantly increase adhesiveness of monocytes to a confluent VEC monolayer and that this occurs under both static [159] and flow [160] conditions.

Complementing these pro-inflammatory, adhesive characteristics of GPR4, in the original report on GPR68 in human VSMCs [163], acidic pH was found to markedly elevate cyclic AMP (likely occurring via Gα_s_/Gα_q/11_) and to increase intracellular Ca^2+^ and prostacyclin production at an acute time point. In another study the long-term effects of extracellular acidification on GPR68 signals in human VSMCs were examined and the authors observed ability of acidic pH to induce COX-2 signaling, prostacyclin production, MAPK phosphatase, and plasminogen activator inhibitor (PAI-1) [161]; however, many of these results including the observation that acidic pH inhibits cell proliferation were believed to be independent of GPR68 [161]. In line with this theory, acidification has, in general, been reported to influence VSM-mediated vessel dilation and growth [164,165], yet comprehensive documentation of precise involvement of GPR68 (or other pH-sensing GPCRs) in the VSM response to acidosis and/or in pathologic vascular growth is lacking.

The potential role of acidosis/pH-sensing heterotrimeric GPCRs in cardiac disease and dysfunction is emerging. Acidosis is a critical byproduct of many cardiac disease states including ischemia and therefore may be a potential target for the treatment of cardiac disorders. Although GPR4 and GPR68 have been identified primarily in VECs and VSMCs, respectively, whether or not these GPCRs are localized to cardiomyocytes remains unclear. Recent investigations have indicated that experimental MI in a murine model results in greatly increased GPR68 expression in cardiomyocytes within the border zone of the infarcted region 7 days following occlusion [166]. These results concur with preliminary experiments in our own lab that show significant (*p* < 0.05) upregulation of GPR68 protein expression (normalized to total protein; n = 4/group) in mouse cardiac homogenates subjected to a 24 h permanent coronary artery ligation compared to naïve control homogenates (data not shown). It is interesting to note that we have not observed upregulation of GPR68 when myocardial ischemia and acidosis has been corrected by reperfusion (I/R; data not shown). Furthermore Russell et al., provided data suggesting that activation of GPR68 following ischemia was responsible for the upregulation of pro-survival and cardioprotective pathways [166]. GPR4 has also been implicated in mediating outcomes following MI, whereby antagonization of GPR4 was able to completely reduce 28-day mortality following a permanent coronary artery occlusion compared to vehicle controls [167]. Despite both GPR4 and GPR68 having apparent roles in the pathophysiology of cardiac ischemia, it may be that they have opposing roles in cardiomyocytes in the presence of ischemic insult. Of course, it is also possible that in the case of GPR4, there may be no direct effect on cardiomyocytes and the observed reduction in mortality observed following inhibition of GPR4 was due to primary effects on VECs within cardiac circulation and a decreased VEC-mediated inflammatory response. The role of acidosis/pH-sensing heterotrimeric GPCRs in cardiac ischemia with the gold standard therapeutic, reperfusion, has yet to be thoroughly investigated and poses additional questions to how to modulate pH-sensing GPCRs in the treatment of cardiac disease.

Another GPCR family that has the capacity to exert significant biological effects on cardiac and vascular tissues and that are dependent in part on cyclic nucleotide-driven protein kinases are the protease-activated receptors (PARs). Extracellular serine proteases serve pivotal roles in many aspects of cardiovascular homeostasis and physiology yet are also involved in the pathogenesis of cardiac and vascular disorders largely through activation of their respective PARs [168,169]. PARs are proteolytically cleaved and activated by these proteases, thereby revealing a new amino-terminus which acts as an intramolecular ligand leading to sustained receptor activation [170]. Following activation, PARs can be rapidly down-regulated by β-arrestin-mediated desensitization and endocytosis followed by lysosomal targeting and degradation [168,169,170]. PARs are normally expressed abundantly in platelets and in relatively low levels in VECs and VSMCs and in cardiac myocytes and fibroblasts [168,171,172]. In VECs PARs operate to regulate vascular tone via induction of NO release and subsequent sGC activation and cyclic GMP/PKG induction [168,173], and in stimulated VSM PARs mediate contraction, migration, proliferation, hypertrophy and ECM production which contribute to the development of vascular lesions and CVD pathogenesis [173,174,175]. Increasing evidence [168,175] supports involvement of PARs in CVD pathophysiology yet their discrete mechanisms have yet to be solidified.

To date four PAR family members have been identified: PAR1, PAR3 and PAR4 are cleaved and activated predominantly by thrombin whereas PAR2 is activated primarily by trypsin and mast cell tryptase [176]. Of these PARs, PAR1 was the first discovered [168] and has since been the most investigated. Early studies identified a role for PAR1 in regulating platelet activation as an underpinning of thrombosis, in turn leading to the creation of Vorapaxar (SCH530348), a selective, competitive antagonist of PAR1. Vorapaxar received FDA approval in 2014 after the Thrombin-Receptor Antagonist in Secondary Prevention of Atherothrombotic Ischemic Events trial (TRA 2°P-TIMI 50) found it to significantly reduce secondary ischemic events compared to placebo controls [177]. In rat VSMCs, PAR1 was shown to be induced following balloon catheter-induced injury [178] and to be upregulated in a hypertensive model [179]. While PAR1 has been more extensively researched as a key player in cardiovascular pathology, recent evidence that PAR2 also plays a regulatory role. PAR2 has been implicated in mediating inflammatory changes in human VSMCs via interaction with soluble dipeptidyl peptidase 4 (DPP4), a ubiquitously expressed cell-surface protease [172], and apolipoprotein E/PAR2 double knockout mice demonstrated significant decreases in atherosclerotic lesion development and aortic inflammatory cytokine release compared to wild-type controls [180]. Preliminary data generated in our lab show upregulation of both PAR2 and PAR4 protein expression as well as phosphorylated Erk1/2 as an indicator of PAR activity in balloon-injured rat carotid arteries compared to uninjured arteries 30 post-injury (data not shown), and in cultured VSMCs activation of PAR2 or PAR4 show co-dependency following pharmacologic activation or inhibition (data not shown). Indeed, the central role of serine proteases and PARs in cardiac and vascular physiology and pathology warrants continued study as plausible clinical targets against CVD.

**Figure 6:**
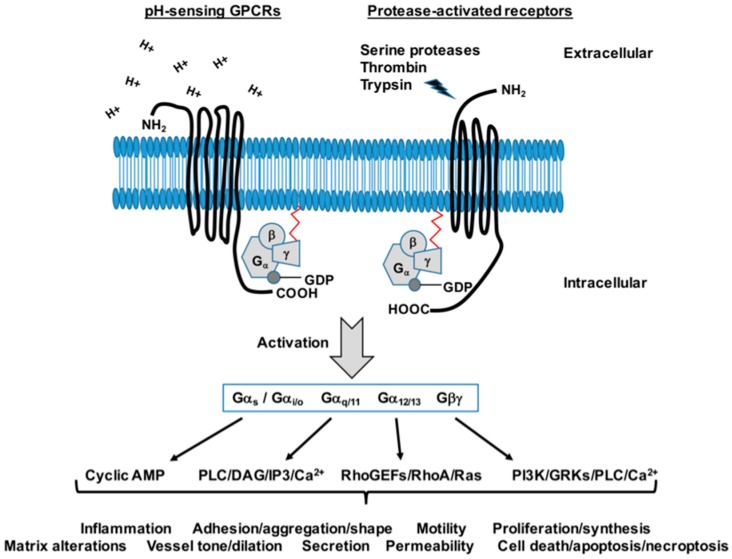
Generalized G protein-coupled receptors: pH-sensing and protease-activated receptors. A schematic depicting a typical pH-sensing GPCR and a protease-activated receptor (PAR) GPCR under non-activated conditions. Both GPCRs are 7 trans-membrane receptors with an extracellular amino terminus and an intracellular carboxyl end associated with G protein subunits. Activation of the pH-sensing GPCR involves extracellular amino terminal histidine sensing of acidic protons (H+) while PAR activation involves cleavage of the extracellular amino terminus by serine proteases, thrombin, trypsin and other agonists and creation of an activating tethered ligand. These GPCRs then stimulate a cascade of G protein-mediated intracellular signals that have the capacity to govern a wide array of inflammation- and growth-regulatory processes in cardiac and vascular tissues.

Regarding mechanisms of cellular signaling, PARs primarily operate via Gα_i/o_ (to inhibit AC and cyclic AMP synthesis), through Gα_12/13_ (to activate RhoGEFS/RhoA/Ras) and via Gα_q/11_ (to moderate PLC/IP_3_/DAG and Ca^2+^ signaling), as well as through dissociated Gβγ (to induce PI3K, GRKs, PLC and Ca^2+^ signaling). These processes then lead to modulation of PKA, PKC and MAPK/ERK pathways, in turn eventuating in the regulation of inflammation- and growth-specific functional processes. A schematic depicting a generic pH-sensing GPCR and PAR as well as some of their activated effectors is shown in Figure 6.

## 8. TGF-β/Smad Signaling

The TGF-β superfamily of growth factors are multifunctional cytokines responsible for regulating key developmental and homeostatic cellular functions such as proliferation, differentiation, recognition, apoptosis, adhesion, and migration and have become of major scientific focus over the past several decades [181]. TGF-β can exert its actions in context-specific fashion, sometimes showing different and even opposite effects in varying cell types and environments. Even though non-Smad pathways exist, one primary route through which TGF-β exerts its functions is through the recruitment and phosphorylation of intracellular Smad proteins [181].

In short, TGF-β-dependent Smad signaling is initiated by binding of a TGF-β ligand (existing in three isoforms: TGF-β1, β2, or β3) to a TGF-β type II receptor (TβR-II), thereby causing a TGF-β type I receptor (TβR-I) to co-localize with the TβR-II. While both receptors contain an intracellular Ser/Thr kinase domain, the kinase domain of TβR-II is constitutively active and its phosphorylation is independent of ligand binding. Once the two receptors become associated, the kinase domain of TβR-II phosphorylates the kinase domain of TβR-I and subsequently transmits the signal through phosphorylation of intracellular Smad [182,183,184]. There are 8 members within the Smad family of proteins that are divided into 3 distinct groups: receptor-regulated Smads (R-Smads), common-mediator Smad (Co-Smad), and inhibitory Smads (I-Smads). The R-Smads (Smads1, 2, 3, 5, and 8, with Smads2 and 3 of primary interest in cardiovascular tissues) are directly phosphorylated by TβR-I and bind with the Co-Smad4 to form a heteromeric complex that can then travel to the nucleus to act as a transcription factor. This process is negatively regulated by inhibitory Smad6 and Smad7 through competing with R-Smads for TβR-I, interacting with Co-Smad4, and/or by initiating the degradation of TGF-β receptors [181].

Recent studies have shown that a correlation exists between the activation of synthetic and growth-promoting TGF-β1, the most abundant and important isoform in the cardiovascular system, and the pathology of CVD [185,186]. Cell-to-cell adhesion through components of the ECM is required for normal growth conditions; however, these adhesive interactions have also been linked to CVD pathogenesis. TGF-β1 is thought to synthesize ECM elements through a Smad3-dependent pathway [187,188,189]. Considering this correlation between TGF-β1 and CVD, studying its regulation and mechanistic effects on cell proliferation and migration could prove beneficial in combatting CVD pathologies. Past studies involving TGF-β1-directed Smad3 have shown conflicting effects of Smad3, reportedly switching between pro-growth and anti-growth phenotypes depending cell type, concentration, and density [190,191].

**Figure 7:**
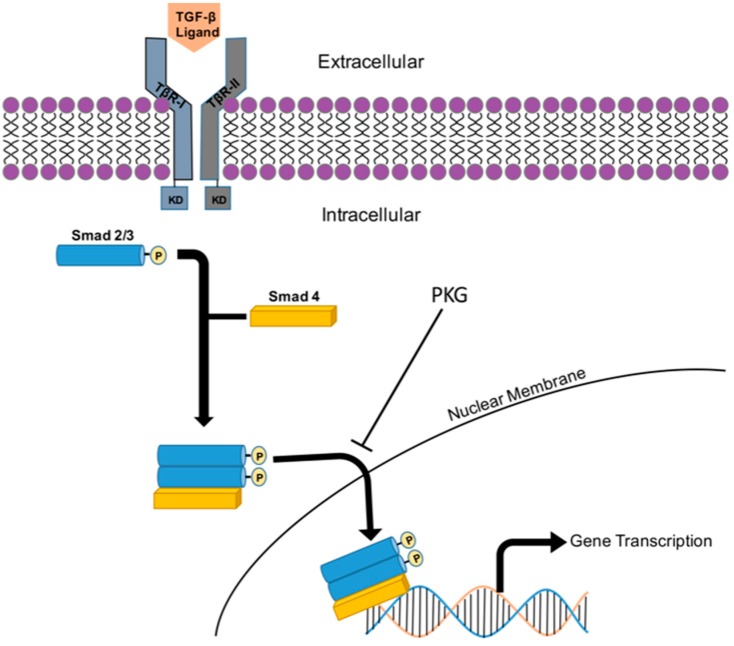
Schematic of transforming growth factor-β (TGF-β) signaling. Binding of an active TGF-β ligand initiates the co-localization of the type II (TβR-II) and type I (TβR-I) TGF-β receptors. The TβR-II remains constitutively phosphorylated and when co-localized phosphorylates the Ser/Thr kinase domain (KD) of the TβR-I. The intracellular signal is subsequently transmitted by phosphorylation of Smad proteins, primarily Smad2/3 in cardiovascular tissues. The phosphorylated Smad proteins combine with Smad4, a common Smad, to form a heterotrimeric complex that can then be shuttled through the nuclear membrane ultimately acting as a transcription factor for inflammatory, synthetic, and growth-promoting genes. The cyclic GMP/PKG system is known to inhibit this pathway by sequestering p-Smad in the cytosol, in turn not allowing it to be shuttled into the nucleus and affect gene transcription.

The impact of TGF-β signaling on cellular dynamics have been shown at least in part to be regulated by cyclic nucleotide-directed protein kinases. A recent study in fibroblasts showed that pretreatment with cyclic GMP in the presence of TGF-β significantly reduced the amount of phosphorylated Smad3 translocated into the nucleus, in turn limiting its transcriptional capacity [192]. A similar result occurred in pulmonary artery VSMCs where translocation of phosphorylated Smad3 into the nucleus was inhibited by the sequestering of phosphorylated Smad3 to cytosolic β2-tubulin via actions of cyclic GMP-directed PKG [193]. Additionally in this study, treatment with a PDE5 led to an increase in the bioavailability of cyclic GMP/PKG and enhanced its inhibition of Smad3 signaling. Following PDE5 inhibition in cells that were pretreated with TGF-β, fibroblast proliferation and alpha smooth muscle actin (α-SMA) were markedly reduced compared to TGF-β only treatment [194]. These findings indicate that cyclic GMP/PKG act to limit the effects of TGF-β signaling by diverting phosphorylated Smad3 away from the nucleus so that it can no longer act, along with Smad2 and Smad4, as a transcription factor and offer support for cyclic GMP/PKG as a transcriptional regulator of Smad-dependent signal transduction. Figure 7 shows a cartoon of TGF-β/Smad signaling and transcriptional control along with depiction of PKG-mediated cytosolic retention of Smad and suppression of these processes.

## 9. FoxO Transcriptional Signaling

A subgroup of the Forkhead family of transcription factors, Forkhead box O (FoxO) proteins, are characterized by their DNA-binding domain consisting of three α-helices and two loops resembling a butterfly wing motif [195]. Only four mammalian FoxO members are currently known, FoxO1, 3, 4, and 6, and these have been implicated in various cellular processes including cell-cycle regulation, differentiation, apoptosis, and oxidative stress response [196,197,198,199]. FoxO transcription factors are tightly regulated by Akt-mediated phosphorylation via the insulin-like growth factor-1 (IGF-1)/phosphatidylinositol-3-kinase (PI3K)/Akt pathway at three conserved amino acid residues [200]. Phosphorylation of FoxO can cause both retention in the cytoplasm or translocation from the nucleus into the cytoplasm, both processes inhibiting it from influencing transcriptional gene regulation and targeting it for degradation [201].

As previously described, VSMC migration and proliferation are key cellular components of CVD. Over the past decades, the impact of FoxO members on cellular dynamics has been studied in relation to CVD pathogenesis. FoxO3a has been implicated in CVD pathology yet the mechanism through which FoxO3 may exacerbate or ameliorate CVD is currently unknown. FoxO3 has been proposed to modulate cell proliferation and cell death pathways [202,203,204]. Overexpression of FoxO3a was shown to increase gene expression of the cell cycle-dependent kinase inhibitor (CdkI) p27 (Kip1), resulting in cell cycle arrest and attenuation of growth in VSMCs and muscle precursor cells [205,206]. Furthermore, cysteine-rich angiogenic protein 61 (Cyr61), a gene expressed immediately following growth factor stimulation, was inhibited by binding of FoxO3 at the Cyr61 promoter region, illustrating another mechanism by which FoxO3 inhibits vascular cell growth [207].

It has been theorized that a dynamic relationship exists between Smad3 and FoxO3 transcription factors. The gene expression of muscle-specific RING finger-1 (MuRF-1), a ubiquitin ligase, was altered by the levels of FoxO3 and/or Smad3 expression [208]. Smad3 binding to the promoter region of MuRF-1 was shown to increase the abundance of FoxO3 bound to that same promoter region and necessary for optimal FoxO3-induced MuRF-1 gene transcription. Additionally, overexpression of Smad3 increased FoxO3 protein abundance and a synergistic effect was observed on FoxO-induced transcription with co-expression of these transcription factors [208]. In endothelial cells, FoxO and Smad synergistically induced expression of the CdkIs p15Ink4b and p21Cip1 [209,210]. Considering both Smad3 and FoxO transcription factors are implicated in the pathogenesis of CVD, further studies aimed at elucidating Smad3/FoxO3 interactions in VSM could prove beneficial in combatting the etiologies associated with CVD.

While the relationship between elements in FoxO signaling and cyclic nucleotide-directed protein kinases has not been thoroughly investigated, FoxO proteins have been associated with the ability of NO to modulate proliferation and migration in damaged VECs [211]. NO-induced inhibition of cell growth occurs through cyclic GMP and PKG, leading to activation of the PI3K/Atk pathway [212,213]. As a result, FoxO, a downstream phosphorylation target of activated PI3K/Atk, is either maintained in the cytoplasm or, if nuclear, is shuttled into the cytoplasm, in turn targeting it for degradation and preventing its growth-inhibitory, cytostatic effects. Further investigation into the regulation of FoxO proteins, their relationship with growth-promoting synthetic Smads, and their control by cyclic nucleotide-driven protein kinases is necessary to understand the mechanisms underlying growth pathologies in cardiovascular tissues.

## 10. Summary & Future Directions

Despite ample basic science and translational investigation, CVD remains the major cause of morbidity and mortality in the United States and worldwide, and all estimates suggest an increasing trend in their prevalence over the next several decades. While some authorities suggest that many of the causes behind CVD are preventable, cumulative efforts must continue in order to gain a more thorough understanding of the key elements that serve as the basis for CVD. Only through these endeavors can we hope to better understand crucial aspects of cardiac and vascular pathologies with the aim of expanding our clinical knowledge and therapeutic efficacy. The cyclic nucleotide-driven protein kinase systems represent wide-ranging and multi-functional processes capable of controlling many mechanisms underlying CVD and can serve as current and emerging targets for intervention. Only through expanded knowledge of these many facets of cyclic nucleotide-driven protein kinases and their diverse downstream effectors including their promiscuous ‘associated kinases’ can we advance our control of these dreaded diseases. In this light, future efforts should be aimed at more completely establishing cyclic nucleotide-driven protein kinases as a therapeutic target to combat and perhaps eliminate CVD, possibly through genetic manipulation/editing or transcriptional control (i.e., Smad/FoxO) of inflammatory/synthetic/growth elements, with pre- and/or post-conditioning interventions [201], or via personalized or precision medicine approaches [3].
